# Psychometric properties and validation of a four-item version of the Strauss–Carpenter scale in bipolar disorder

**DOI:** 10.1186/s40345-016-0063-3

**Published:** 2016-10-18

**Authors:** Susana Alberich, Sara Barbeito, Itxaso González-Ortega, Amaia Ugarte, Patricia Vega, Sonia Ruiz de Azúa, Purificación López, Iñaki Zorrilla, Ana González-Pinto

**Affiliations:** 1 Department of Psychiatry, Bioaraba Research Institute, Araba University Hospital, Olaguibel Street 29, 01004 Vitoria, Spain; 2 Biomedical Research Networking Centre in Mental Health (CIBERSAM), OSI Araba-University Hospital, Vitoria, Spain; 3 Grupo de Investigación en Psicología clínica de la Unir, Madrid, Spain; 4 University of the Basque Country, Vitoria, Spain

**Keywords:** Strauss–Carpenter, Functioning, Bipolar, Prognosis, Outcome

## Abstract

**Background:**

Bipolar disorder is a chronic illness that impairs functioning and affects the quality of life of patients. The onset of this illness usually occurs at an early age, and the risk of relapse remains high for decades. Thus, due to the great clinical relevance of identifying long-term predictors of functioning in bipolar disorder, Strauss and Carpenter developed a scale composed of items known to have prognostic value.

**Methods:**

To determine the clinical usefulness of the four-item Strauss–Carpenter scale in bipolar disorder, a 1-year prospective follow-up study was carried out. The internal consistency, convergent and discriminant validity, and test–retest reliability of the scale were assessed. We also compared the Strauss–Carpenter scale with the reference scales Global Assessment Functioning (GAF), Clinical Global Impression for Bipolar Disorder, the Modified Version (CGI-BIP-M) and the Sheehan Disability Scale (Sheehan). Additionally, a cut-off point for remission was established.

**Results:**

The total sample was composed of 98 patients with a diagnosis of bipolar disorder. The four-item version of the Strauss–Carpenter scale showed to have appropriate psychometric properties, comparable to those of reference scales. The best cut-off point for remission was 14.

**Conclusions:**

The four-item version of the Strauss–Carpenter scale has suitable validity and reliability for the assessment of functioning in patients with bipolar disorder.

## Background

Bipolar disorder is a chronic illness that affects functioning and the quality of life of patients both, during manic and depressive episodes and during remission (Rosa et al. 2008; Burdick et al. 2010; Jiménez et al. 2012; Cotrena et al. 2015). Patients show different clinical characteristics, severity, comorbidities and treatment response. The prevalence of bipolar disorder I and II ranges from 2 to 4 % (Kessler et al. 1994, 2005, 2012).

Since the onset of bipolar disorder usually occurs at an early age and the risk of relapse persists for decades until the age of 70 years (Angst et al. 2002), the identification of long-term predictors of bipolar disorder risk is highly relevant to the clinical management and treatment of patients, as well as to the development of preventive strategies (Pinna and Manchia 2014).

In a longitudinal study, Strauss and Carpenter (1972) designed a four-item scale based on four areas of outcome dysfunction which had been used as criteria of outcome in other studies. The authors demonstrated that this scale was effective in predicting outcomes in schizophrenia with a follow-up of 2 years. Some decades later, Poirier et al. (2004) validated the French translation of a revised version of the latter scale in patients with schizophrenia (SCOCS-R). The scale showed a high reliability and validity. Nieman et al. (2013) analyzed an extended version of the Strauss–Carpenter scale (Strauss and Carpenter 1974) and found that it was effective in predicting transition to a first psychotic episode in patients at high risk of psychosis. This scale has been used in many studies on first psychotic episodes that included patients diagnosed either with schizoaffective disorder or bipolar disorder (Melle et al. 2000; Castro-Fornieles et al. 2011; Evensen et al. 2012; Barbeito et al. 2014; Jordan et al. 2014; Parellada et al. 2015). However, although the scale has been translated into Spanish for its use in schizophrenia (Ahuir et al. 2009), it has not been translated yet for its use in bipolar disorder, despite that Spanish is the second-most spoken language in the world. The objective of this study was to assess the reliability and validity of the four-item Strauss–Carpenter prognostic scale in measuring functioning in patients with bipolar disorder in the Spanish population.

## Methods

### Design

A 1-year follow-up longitudinal study was performed to validate a brief version (four items) of the Strauss–Carpenter scale for patients with bipolar disorder. The reliability, internal consistency, convergent and predictive validity, and prognostic capacity of the scale were assessed. To such purpose, the level of correlation between the score obtained on the four-item scale and two reference variables of functioning was evaluated.

### Subjects

A total of 98 patients formed the final sample of the study. All subjects were aged between 18 and 65 years and met the diagnostic criteria for bipolar disorder type I as assessed by the Structured Clinical Interview for The Diagnostic for the Statistical Manual of Mental Disorders, 4th edition (DSM-IV), Axis I Disorders (SCID-I) (American Psychiatric Association 1994). The study was carried out at Araba University Hospital, Vitoria, Spain. All patients were included after informed consent for participation was obtained. The exclusion criteria were: organic brain disorder, comorbidity with organic mental retardation or clinical decompensation requiring hospitalization in an acute inpatient unit.

### Measures

The original scale was translated from English to Spanish by two independent bilingual translators who were familiar with the content and purposes of the scale. Each of them made a forward translation of the scale and then both translations were merged into the final version of the scale. Subjects were evaluated using a protocol that included the following scales: the Strauss–Carpenter prognostic scale (Strauss and Carpenter 1972), was employed to assess the psychosocial functioning of patients. This scale consists of four items rated from 0 to 4 on a Likert-type scale and yields a total score that is calculated by the addition of all item scores: the higher the score, the better is the prognosis; Global Assessment Functioning (GAF) (Endincott et al. 1976), which also evaluates the general functioning of patients and in which a higher score indicates better functioning; the Clinical Global Impression for Bipolar Disorder, Modified Version (CGI-BIP-M) (Vieta et al. 2002), which assesses the severity of the disease; and the Sheehan Disability Scale (Sheehan et al. 1996), which assesses functional impairment in patients. The interpretation of the two latter scales is inverse; therefore, higher scores indicate higher gravity. All subjects were evaluated using this protocol at baseline and at 1-year follow-up.

Other relevant clinical and socio-demographic variables were also collected, such as age, gender, civil status, educational level, suicide attempts, substance use or number of hospitalizations and episodes in the last year.

The study was approved by the Ethics and Research Committee of the Araba University Hospital.

### Statistical analysis

The internal consistency of the Strauss–Carpenter scale was examined by assessing the homogeneity of items using Cronbach’s alpha.

Convergent validity was calculated using Pearson’s correlation coefficient between the total score on Strauss–Carpenter scale and scores on the reference scales at baseline (GAF and Sheehan), as we considered they were continuous variables. The Spearman correlation was used to assess correlations with the CGI-BIP-M scale, as it has an ordinal measure. The predictive validity of the scale was assessed by calculating Spearman correlations between items of the Strauss–Carpenter scale (since they have an ordinal measure) and the reference scales at 1 year. In the case of the total score on the Strauss–Carpenter scale, its relation with the GAF and Sheehan scales was assessed by Pearson correlations. Consistency between values and test–retest reliability of the Strauss–Carpenter was evaluated by comparing baseline and 1-year values by intra-class correlation coefficients (ICC).

Finally, ROC curves were used to evaluate the discriminant capacity of the scale. The area under the curve (AUC) and cutoff point for remission were also determined.

All statistical analyses were performed using the IMB SPSS statistical software package versions 23 and R 3.1.2 (R Core Team 2014).

## Results

### Socio-demographic data

Of the 98 patients included in the sample, men accounted for 66.3 % of the sample, and the mean age was 29.38 (8.11) years. Most subjects were single (85.4 %) and had primary education (41.2 %); seven (7.5 %) had attempted suicide. Regarding substance use, 27.7 % used alcohol, 48 % smoked cannabis and 28.9 % took other drugs (Table 1).

**Table 1:** Socio-demographic data at baseline (*n* = 98)

Variables	Categories	*n* (%), means (SD)
Gender	Men	65 (66.3 %)
	Women	33 (33.7 %)
Age		29.38 (8.11)
Civil status	Single	82 (85.4 %)
	Married	10 (10.4 %)
	Other	4 (4.2 %)
Educational level	Without studies	0 (0 %)
	Primary education	40 (41.2 %)
	Secondary education	32 (33 %)
	University	25 (25.8 %)
Alcohol		13 (27.7 %)
Cannabis		47 (48 %)
Other drugs		28 (28.9 %)
Suicide attempts		7 (7.5 %)
Previous episodes		1.25 (0.59)
Previous hospitalizations		1.10 (0.42)

### Psychometric characteristics

#### Internal consistency

Cronbach’s alpha for the items of the Strauss–Carpenter scale was 0.677.

#### Convergent validity

Pearson correlation between the total score on Strauss–Carpenter and the reference scales was significant and in the expected direction, both, for the CGI-BP-M and the Sheehan scale (*p* < 0.001) (Table 2).

**Table 2:** Convergent validity of the four-item Strauss–Carpenter scale

Reference scales	Strauss–Carpenter scale	
	Pearson’s *r* coefficient	*p* value
GAF	0.087	0.339
CGI-BIP-M	−0.668^a^	<0.001
Sheehan Disability Scale	−0.871	<0.001

^a^ Spearman’s correlation coefficient *rho* is shown for the CGI-BIP-M scale

#### Test–retest reliability

Test–retest reliability was calculated using ICC. The total score on Strauss–Carpenter was found to have a good intra-class correlation (Table 3).

**Table 3:** Reliability analysis

Strauss–Carpenter scale	Intraclass correlation coefficient (ICC)		
	Coef	*p*	95 % CI
Item 1. Hospitalization	0.424	0.004	0.140–0.614
Item 2. Work	0.648	<0.001	0.475–0.764
Item 3. Social activity	0.609	<0.001	0.546–0.796
Item 4. Symptoms	0.588	<0.001	0.384–0.724
Strauss–Carpenter total score	0.749	<0.001	0.626–0.832

#### Predictive validity

Table 4 shows the correlation between the Strauss–Carpenter scale (both of each item separately and of the total score) and 1-year values on the reference scales. A significant correlation was found between the Social Activity item (Item 3) and the total score on the Strauss–Carpenter scale and the three reference scales. The strongest correlation was observed with the Sheehan scale (rho = −0.50 and *r* = −0.57, respectively). The Hospitalization item (Item 1) and the Symptoms item (Item 4) showed to be significantly correlated with both CGI-BIP-M and Sheehan. Of note, the Hospitalization item was more strongly correlated with the CGI-BIP-M scale (rho = −0.37), whereas the Symptoms item was more significantly correlated with the Sheehan scale (rho = −0.48). Finally, a significant relationship was observed between the Work item (item 2) and GAF and Sheehan scale, but not with the CGI-BIP-M. Again, the strongest correlation was observed with the Sheehan scale (rho = −0.38) (Table 4).

**Table 4:** Correlations between Strauss and Carpenter and the reference scales

Strauss–Carpenter	GAF		CGI-BIP-M		Sheehan	*p*
	rho	*p*	rho	*p*	rho	
Item 1. Hospitalization	0.150	0.141	*−0.365*	*0.012*	*−0.329*	*0.001*
Item 2. Work	*0.268*	*0.008*	−0.155	0.127	*−0.381*	*<0.001*
Item 3. Social activity	*0.254*	*0.012*	*−0.413*	*0.002*	*−0.500*	*<0.001*
Item 4. Symptoms	0.189	0.063	*−0.469*	*<0.001*	*−0.476*	*<0.001*
Strauss–Carpenter total score	*0.337* ^a^	*0.001*	*−0.507*	*<0.001*	*−0.573* ^a^	*<0.001*

Italic values indicate the values reaching statistical significance (*p* < .05)

^a^ Pearson’s correlation coefficient *r* is shown for correlation between the total score of the Strauss–Carpenter and GAF and Sheehan scales

#### Discriminant capacity

**Fig. 1:**
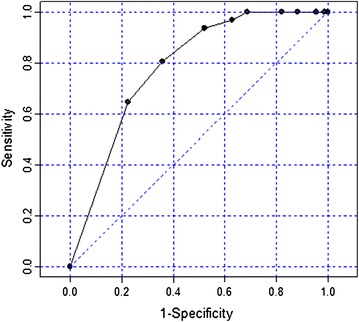
ROC curve of the four-item Strauss–Carpenter scale

The discriminant capacity of the four items of the Strauss–Carpenter scale was assessed using ROC curves. The area under the curve (AUC) was 0.784 (95 % CI 0.695–0.874), which indicates a good discriminant capacity, as it is close to 1, the maximum value (Fig. 1). Moreover, the best correlation between sensitivity and specificity (70 and 64.2 %, respectively) was obtained using a cutoff point of 14 in the total score on the four-item Strauss–Carpenter scale.

## Discussion

Currently, most instruments for assessing functional impairment in patients with bipolar disorder are based on global measures. The global assessment functioning (GAF) (Endincott et al. 1976) is the most commonly used tool for the evaluation of functioning. Nevertheless, several studies suggest that this scale might be mediated by symptoms (Samara et al. 2014; Suzuki et al. 2015).

There are few instruments that assess different areas of impairment and also have a prognostic value. The Functioning Assessment Short Test (FAST) validated by Rosa et al. (2007) is divided into six specific areas of functioning: Autonomy, Occupational Functioning, Cognitive Functioning, Finances, Personal Relationships and Leisure. This scale showed strong psychometric properties in the assessment of cognitive impairment in patients with bipolar disorder. Poirier et al. (2004) validated the translation of a revised version of the Strauss–Carpenter scale in schizophrenia. This version consisted of nine items and showed high reliability and validity. Ahuir et al. (2009) analyzed an extended version of the Strauss–Carpenter scale (Strauss and Carpenter 1974) in schizophrenia and obtained high values of validity and reliability. This confirmed its good predictive properties. However, no reliable and valid instrument has been designed yet that assesses functioning and also has a prognostic value in bipolar disorder.

The results obtained in this study showed that the four-item Strauss–Carpenter scale for patients with bipolar disorder have adequate psychometric characteristics for this population, both in terms of reliability and validity. Therefore, this is an adequate instrument with discriminant and prognostic capacity.

Regarding the internal consistency of the scale, although a psychometric instrument is generally considered reliable if Cronbach’s *α* > 0.70 (Bland and Altman 1997), in this study we have obtained a value of 0.677, which approaches this limit to be considered acceptable. Besides, it must be considered that this coefficient is affected by the length of the scale (Streiner 2003).

With regard to convergent validity, we did not observe a significant correlation between the Strauss–Carpenter and the GAF scale, although the CGI-BIP-M and the Sheehan scale were found to be strongly correlated. Further, as expected, these correlations were inverse.

Ahuir et al. (2009) also demonstrated that the 17-item version of the Strauss–Carpenter scale has a high convergent validity. In this case, the scale correlated significantly with the CGI, the World Health Organization Disability Assessment Schedule (WHO-DAS), the Positive And Negative Syndrome Scale (PANSS) and the Satisfaction With Life Domains Scale (SLDS), and also with the GAF.

Poirier et al. (2004) also obtained a high convergent validity although, in this case, correlation was with the Social and Occupational Functioning Assessment Scale (SOFAS).

In the analysis of the test–retest reliability, the items and total score on the Strauss–Carpenter scale showed adequate and high intra-class correlation coefficients (except for the hospitalization item). This confirms the stability and consistency of the first assessment of the test. The result for the Hospitalization item could be explained because one of the exclusion criteria was that patients were not hospitalized at recruitment, but they could have been hospitalized during follow-up. Thus, this item could have more variability in the test–retest.

Regarding the predictive validity of the four-item scale, the items with the best prognostic value were those that assess social activity and symptoms (Items 3 and 4), which showed a high correlation with the reference scales. The total score on Strauss–Carpenter was also significantly related to the three reference scales, showing a remarkably close relationship with the Sheehan scale (*r* = −0.573). As expected, this correlation supports the hypothesis on the predictive value of the scale. This agrees with the results published by Ahuir et al. (2009), who observed a significant correlation (*p* < 0.01) between Strauss and Carpenter and GAF, CGI and WHO-DAS.

Finally, the ROC curves confirmed that the four-item Strauss–Carpenter scale has a high discriminant capacity, with an area under the curve of 0.874. Moreover, we found that a cutoff point of 14 optimizes sensitivity (the probability that the test correctly detects subjects with a poor prognosis of functioning) and specificity (the probability that the test correctly detects subjects with a good prognosis of functioning).

This study has some limitations such as the small sample size and the short duration of follow-up (a year). Future studies should analyze the psychometric properties of the four-item Strauss–Carpenter scale in larger epidemiological samples and with a longer follow-up. Another limitation is that there was no control group in this study. Future studies should include a control group to confirm the results of this study.

## Conclusions

The adaptation of the four-item Strauss–Carpenter scale to patients with bipolar disorder has adequate psychometric properties and is an acceptable and very useful instrument, not only for it shortness, but also for its prognostic capacity. This could have great clinical relevance for the clinical and pharmacological management of patients. In addition, the use of this tool facilitates earl intervention to prevent the unfavorable evolution of patients with a worse prognosis.

## Abbreviations

SCOCS-R: Strauss and Carpenter revised outcome criteria scale
GAF: Global Assessment Functioning
CGI-BIP-M: Clinical Global Impression for Bipolar Disorder Modified Version
ICC: intra-class correlation coefficient
AUC: area under the curve
FAST: Functioning Assessment Short Test
WHO-DAS: World Health Organization Disability Assessment Schedule
PANSS: Positive and Negative Syndrome Scale
SLDS: Satisfaction with Life Domains Scale
SOFAS: Social and Occupational Functioning Assessment Scale
